# Trends in body mass index among Chinese children and adolescents aged 7–15, 2010–2022

**DOI:** 10.3389/fped.2026.1976436

**Published:** 2026-10-28

**Authors:** Haodong Zhong, Tao Tao, Zhiyuan Hei

**Affiliations:** 1 School of Marxism, Yueyang Vocational College of Science and Technology, Yueyang, China; 2 Hunan Sports Vocational College, Changsha, China; 3 Department of Physical Education and Health Teaching and Research, Hunan Applied Technology College, Changde, China

**Keywords:** body mass index, children and adolescents, China family panel study, obesity, overweight, trends

## Abstract

**Purpose:**

To characterize trends in body mass index (BMI) and in overweight and obesity prevalence among Chinese children and adolescents aged 7–15 from 2010 to 2022, and to inform targeted weight-management strategies.

**Methods:**

Data from 27,755 person-observations drawn from seven waves (2010–2022) of the China Family Panel Studies were stratified by sex and age. Overweight and obesity were classified using the sex- and age-specific BMI cut-offs of the Chinese Guidelines for Weight Management (2024 Edition). Mean BMI was summarized by descriptive statistics; trends were assessed with Spearman correlation between survey year and mean BMI, and prevalence differences were compared with chi-square tests; mean annual percentage-point changes between adjacent surveys were also computed.

**Results:**

Body mass index (BMI) at ages 7–15 showed no significant overall linear trend (*P* > 0.05), but increased with survey year among boys aged 10 and older (r_s = 0.082–0.189, *P* < 0.01) and girls aged 11 and older (r_s = 0.079–0.197, *P* < 0.001). Overweight prevalence rose markedly among boys aged 8 and 13–15 and 15-year-old girls (*P* < 0.05), while obesity fell among the youngest boys and girls but rose among boys aged 12–13 and girls aged 13–15 (*P* < 0.05); mean annual percentage-point changes peaked during 2016–2018 and were higher at ages 10–15 than at ages 7–9.

**Conclusions:**

From 2010 to 2022 the prevalence of overweight and obesity diverged by age, improving among younger children but worsening among adolescents, particularly boys aged 10 and older and girls aged 12 and older. Intervention efforts should be brought forward to the upper elementary school years, be differentiated by sex and age, and prioritize weight management for adolescent boys. These trends are descriptive; the observational design precludes causal inference.

## Introduction

Being overweight or obese in childhood and adolescence is well established to elevate the long-term risk of chronic diseases such as cardiovascular disease, diabetes, and dyslipidemia. Currently, the problem of overweight and obesity among children and adolescents is becoming increasingly prominent worldwide and has become a major public health challenge. Data from the World Health Organization indicates that the number of overweight or obese children and adolescents aged 5 to 19 globally has exceeded 340 million. With China's rapid economic and social progress and changing lifestyles, the prevalence of overweight and obesity among children and adolescents has continued to rise. According to publicly available data from the National Health Commission, the prevalence of overweight and obesity among children and adolescents aged 6–17 in China has approached 20%, and the trend is shifting toward younger age groups; obesity has become one of the major public health issues threatening the physical and mental health of China's children and adolescents. In response, the ‘Healthy China 2030’ Planning Outline, issued by the Central Committee of the Communist Party of China and the State Council in 2016, explicitly called for establishing and improving a nutrition monitoring system for the population and strengthening the prevention and control of common diseases among students, such as myopia and obesity; In 2020, the ‘Implementation Plan for the Prevention and Control of Obesity Among Children and Adolescents,’ jointly issued by six departments including the National Health Commission and the Ministry of Education, explicitly called for monitoring the weight and growth and development of children and adolescents, as well as strengthening weight management and interventions for obese children; In 2024, the General Office of the National Health Commission issued the ‘Guiding Principles for Weight Management,’ which explicitly called for the implementation of standardized management of weight-related issues such as overweight and obesity. The document covers interventions and treatments for overweight and obesity, as well as the management and maintenance of a healthy weight. It is evident that this series of policies provides macro-level guidance for weight management among children and adolescents. Body Mass Index (BMI) is a core indicator for assessing weight status and is widely used in research on the growth and development of children and adolescents, as well as in the monitoring of overweight and obesity. Current academic research on BMI largely draws on data from the National Student Physical Fitness and Health Survey and the China Health and Nutrition Survey. However, further research is needed to examine the characteristics of BMI and changes in overweight and obesity among children and adolescents aged 7–15 across gender and age dimensions during the period from 2010 to 2022. as BMI undergoes dynamic changes during the critical growth and development period of ages 7–15 and is influenced by factors such as gender, age, and socioeconomic status; cross-sectional data alone cannot adequately explain its long-term trends. Based on this, this study utilized physical data from 27,755 children and adolescents aged 7–15 collected between 2010 and 2022 in the China Family Panel Studies (CFPS), to analyze the long-term trends in mean BMI and the prevalence of overweight and obesity among children and adolescents aged 7–15. By conducting differentiated analyses based on gender and age, this study aims to identify key age periods during which the risk of abnormal weight increases most rapidly, with the goal of providing empirical evidence to support relevant authorities in managing the weight of children and adolescents and implementing physical education and health interventions in schools.

Although previous nationally representative studies have primarily drawn on the China National Survey on Students’ Constitution and Health or the China Health and Nutrition Survey, the multi-wave CFPS data used here offer a distinctive advantage in that they permit consistent, age- and sex-stratified tracking of BMI and weight status across seven survey years (2010–2022) within a single harmonized panel. The novelty of this study lies in: quantifying age- and sex-specific BMI and overweight/obesity trends across the full 7–15-year span; separating divergent patterns among younger children from those in adolescence; linking these patterns to the policy and behavioral context. This study therefore addresses the following research questions: RQ1—How did BMI and the prevalence of overweight and obesity among Chinese children and adolescents aged 7–15 change between 2010 and 2022, and do the trends differ by sex and age group? RQ2—At what age does the risk of abnormal weight gain accelerate most markedly, and is the timing sex-specific? RQ3—How do the average annual percentage-point changes in overweight and obesity prevalence differ between younger (7–9 years) and older (10–15 years) age groups across the seven survey waves?

## Materials and methods

### Materials

The United Nations Convention on the Rights of the Child defines a child as any person under the age of 18, while organizations such as the World Health Organization, UNICEF, and the United Nations Population Fund define adolescents as those aged 10–19; the United Nations General Assembly defines youth as those aged 15–24. The sample of 7- to 15-year-olds selected for this study fully covers the late childhood stage (ages 7–9) as well as the entire early to mid-adolescence period (ages 10–15), and naturally transitions to the onset of young adulthood (age 15). This age group is in a critical period of growth and development and is a key population for research on dynamic changes in body mass index (BMI) and health interventions. Based on this, this study utilized data on children and adolescents aged 7–15 from the China Family Panel Studies (CFPS) conducted between 2010 and 2022.

### Data filtering

The data for this study were drawn from seven waves of the China Family Panel Studies conducted between 2010 and 2022. Guided by the objectives of the adolescent health study, standard screening procedures were applied to the raw data. The sample was first restricted to children and adolescents aged 7–15 according to the child's age in years. Cases with missing data on gender, height, weight, or the derived BMI were then excluded to ensure the completeness and validity of the key variables. Finally, to eliminate outliers, physiologically implausible height and weight measurements—specifically those outside the range of 0.8–2.2 m for height or 15–150 kg for weight—were removed. Following these procedures, 30,659 child-year records with available age data were initially extracted across the seven waves, and 27,755 valid person-observations remained after exclusions. Because CFPS is a rotating panel in which participants may appear in more than one wave, the data constitute a repeated cross-sectional unbalanced panel rather than 27,755 individuals followed continuously; the analytical sample is therefore reported in person-observations rather than unique individuals. The height and weight thresholds were chosen to reflect physiologically plausible values for children and adolescents aged 7–15 and to remove impossible outliers, and no additional extreme-value algorithm was applied. A participant-flow summary of the records excluded at each screening stage is provided in Table 1. The final analytical sample comprised 27,755 person-observations, including 13,346 males (48.09%) and 14,409 females (51.91%); gender composition differed significantly across survey years (*χ*^2^ = 62.321, *P* < 0.01), as shown in Table 2.

**Table 1:** Participant flow through the data-filtering process, by CFPS wave.

Screening stage	2010	2012	2014	2016	2018	2020	2022	Total
Records extracted (ages 7–15)	5,021	4,655	4,562	4,246	4,587	3,885	3,703	30,659
Excluded^a^	402	691	261	155	256	552	587	2,904
Final valid person-observations	4,619	3,964	4,301	4,091	4,331	3,333	3,116	27,755

a Excluded for missing gender, height, weight, or BMI, or for physiologically implausible height (<0.8 m or >2.2 m) or weight (<15 kg or >150 kg).

**Table 2:** Age and gender distribution of children and adolescents aged 7–15, 2010–2022 (*N* = 27,755).

Age (years)	Male							Female							*χ* ^2^	*P*-value
	2010	2012	2014	2016	2018	2020	2022	2010	2012	2014	2016	2018	2020	2022		
7	202	218	240	255	281	178	154	242	252	271	321	281	204	168	4.275	0.64
8	220	247	253	237	288	188	206	271	260	245	289	224	232	204	21.335	0.002
9	221	187	226	235	292	195	152	251	224	270	258	249	216	188	11.619	0.071
10	264	191	243	200	265	157	179	256	239	284	221	235	206	209	13.242	0.039
11	246	184	196	212	252	183	205	284	209	229	255	214	221	193	12.661	0.049
12	235	223	208	210	239	166	135	275	204	255	253	213	179	185	15.232	0.019
13	250	208	190	171	249	167	169	256	269	221	189	201	190	180	14.501	0.025
14	289	188	242	174	246	163	153	253	222	236	245	215	169	151	19.086	0.004
15	297	232	237	185	199	150	119	307	207	255	181	188	169	166	10.482	0.106
Total	2224	1878	2035	1879	2311	1547	1472	2395	2086	2266	2212	2020	1786	1644	62.321	<0.01

### Statistical analysis

This study aims to analyze the trends in BMI changes among children and adolescents aged 7–15 from 2010 to 2022, as well as the characteristics of changes in the prevalence rates of overweight and obesity across different gender and age groups and their average annual percentage-point changes. It systematically presents the long-term trends in the weight status of this population, using the BMI screening thresholds for overweight and obesity—categorized by gender and age—for children and adolescents aged 7–15 as outlined in the *Guidelines for Weight Management (2024 Edition) (1), which specify BMI cutoff values for overweight and obesity screening by gender and age for children and adolescents aged 7–15, to assess the weight status of the study participants (see Table 3). Specifically, the BMI of individuals in each age and gender group was compared with the corresponding overweight and obesity cutoff values in the table. If a participant's BMI was below the overweight cutoff, they were classified as normal (marked as 0); individuals whose BMI falls within the range between the overweight and obesity thresholds are classified as overweight (marked as 1), and those with a BMI equal to or greater than the obesity threshold are classified as obese (marked as 2). This approach is used to analyze the detection of overweight and obesity. Regarding statistical analysis methods, descriptive statistics were used to calculate the mean and standard deviation of BMI for male and female children and adolescents in each age group for each year, to present the distribution and dynamic changes in BMI; Spearman's rank correlation was computed between the survey year and the age- and sex-specific mean BMI to assess the overall monotonic trend across the seven waves; because Spearman's coefficient reflects a global monotonic association rather than year-by-year change, statistical significance should be interpreted together with the magnitude of r_s; the *χ*^2^ values and *P*-values reported in Tables 4–7 were derived from Pearson's chi-square tests comparing the 2010 and 2022 surveys; We then calculated the average annual percentage-point change of overweight and obesity prevalence for each age group, defined as: average annual percentage-point change = (prevalence rate in the later survey year—prevalence rate in the previous survey year)/number of years between surveys. Because prevalence was already expressed as a percentage, the resulting difference is reported in percentage points per year; the  × 100% term has been omitted to avoid double scaling, to illustrate the trends in these changes. All analyses were performed on unweighted observations; the sampling weights provided by CFPS were not applied (see Limitations). Statistical analyses were conducted using IBM SPSS Statistics version 26.0.

**Table 3:** BMI cutoff values for screening for overweight and obesity by gender and age among children and adolescents aged 7–15.

Age (years)	Overweight Male (kg/m^2^)	Obese Male (kg/m^2^)	Overweight Female (kg/m^2^)	Obese Female (kg/m^2^)
7	17.0	18.7	16.8	18.5
8	17.8	19.7	17.6	19.4
9	18.5	20.8	18.5	20.4
10	19.2	21.9	19.5	21.5
11	19.9	23.0	20.5	22.7
12	20.7	24.1	21.5	23.9
13	21.4	25.2	22.2	25.0
14	22.3	26.1	22.8	25.9
15	22.9	26.6	23.2	26.6

**Table 4:** Prevalence of overweight among male children and adolescents by age group, 2010–2022 *n* (%) (χ^2^ and *P*-values are from Pearson's chi-square tests comparing the 2010 and 2022 surveys.).

Age (years)	2010	2012	2014	2016	2018	2020	2022	χ^2^	*P*-value
7	30 (14.9)	32 (14.7)	22 (9.2)	33 (12.9)	45 (16.0)	31 (17.4)	19 (12.3)	0.28	0.598
8	15 (6.8)	14 (5.7)	23 (9.2)	20 (8.4)	35 (12.2)	19 (10.1)	20 (9.7)	4.57	0.033*
9	33 (14.9)	22 (11.8)	20 (8.8)	26 (11.1)	54 (18.5)	22 (11.3)	12 (7.9)	0.30	0.584
10	25 (9.5)	22 (11.6)	39 (16.1)	17 (8.5)	46 (17.4)	20 (12.7)	27 (15.1)	3.40	0.065
11	25 (10.2)	28 (15.3)	22 (11.2)	22 (10.5)	51 (20.2)	22 (12.0)	23 (11.2)	0.58	0.448
12	28 (12.0)	27 (12.1)	27 (13.0)	16 (7.6)	47 (19.7)	29 (17.5)	10 (7.4)	0.62	0.431
13	14 (5.6)	18 (8.7)	17 (9.0)	21 (12.3)	41 (16.5)	25 (15.0)	24 (14.2)	16.73	<0.001***
14	19 (6.6)	15 (8.0)	14 (5.8)	16 (9.2)	34 (13.8)	19 (11.7)	20 (13.1)	10.86	0.001**
15	16 (5.4)	15 (6.5)	18(7.6)	12(6.5)	24(12.1)	18(12.0)	8(6.7)	5.05	0.025*

**Table 5:** Prevalence of overweight among female children and adolescents by age group, 2010–2022 *n* (%) (χ^2^ and *P*-values are from Pearson's chi-square tests comparing the 2010 and 2022 surveys.).

Age (years)	2010	2012	2014	2016	2018	2020	2022	χ^2^	*P*-value
7	35 (14.5)	39 (15.5)	32 (11.8)	50 (15.6)	39 (13.9)	26 (12.7)	22 (13.1)	0.31	0.580
8	29 (10.7)	29 (11.2)	27 (11.1)	32 (11.1)	28 (12.5)	24 (10.3)	25 (12.3)	0.15	0.694
9	28 (11.2)	28 (12.5)	26 (9.6)	26 (10.1)	15 (6.0)	22 (10.2)	22 (11.7)	0.59	0.444
10	32 (12.5)	31 (13.0)	44 (15.6)	19 (8.6)	31 (13.2)	24 (11.7)	21 (10.0)	1.20	0.274
11	29 (10.2)	23 (11.0)	23 (10.0)	28 (11.0)	20 (9.3)	21 (9.5)	27 (14.0)	0.37	0.542
12	22 (8.0)	19 (9.4)	27 (10.6)	17 (6.7)	19 (8.9)	20 (11.2)	18 (9.7)	0.43	0.514
13	16 (6.2)	25 (9.3)	14 (6.3)	17 (9.0)	18 (9.0)	21 (11.1)	19 (10.6)	3.29	0.070
14	12 (4.7)	9 (4.1)	20 (8.5)	25 (10.2)	13 (6.0)	19 (11.2)	9 (6.0)	3.27	0.071
15	13 (4.2)	14 (6.8)	10(3.9)	16(8.8)	15(8.0)	21(12.4)	20(12.0)	15.77	<0.001***

**Table 6:** Prevalence of obesity among male children and adolescents by age group, 2010–2022 *n* (%) (χ^2^ and *P*-values are from Pearson's chi-square tests comparing the 2010 and 2022 surveys.).

Age (years)	2010	2012	2014	2016	2018	2020	2022	χ^2^	*P*-value
7	48 (23.8)	56 (25.7)	59 (24.7)	71 (27.8)	77 (27.4)	31 (17.4)	21 (13.6)	5.06	0.025*
8	44 (20.0)	66 (26.9)	66 (26.3)	59 (24.9)	66 (22.9)	35 (18.6)	26 (12.6)	7.64	0.006**
9	27 (12.2)	36 (19.4)	42 (18.6)	39 (16.6)	64 (21.9)	24 (12.4)	15 (9.9)	0.41	0.520
10	21 (8.0)	23 (12.1)	31 (12.8)	24 (12.1)	57 (21.5)	24 (15.3)	14 (7.8)	3.09	0.079
11	17 (6.9)	9 (4.9)	14 (7.1)	15 (7.1)	35 (13.9)	13 (7.1)	14 (6.8)	1.47	0.225
12	11 (4.7)	10 (4.5)	7 (3.4)	10 (4.8)	27 (11.3)	11 (6.6)	17 (12.6)	12.89	<0.001***
13	2 (0.8)	7 (3.4)	6 (3.2)	4 (2.3)	16 (6.4)	8 (4.8)	8 (4.7)	7.85	0.005**
14	6 (2.1)	6 (3.2)	1 (0.4)	3 (1.7)	10 (4.1)	4 (2.5)	6 (3.9)	1.86	0.173
15	3 (1.0)	1(0.4)	4(1.7)	3(1.6)	3(1.5)	3(2.0)	2(1.7)	1.29	0.256

**Table 7:** Prevalence of obesity among female children and adolescents by age group, 2010–2022 *n* (%) (χ^2^ and *P*-values are from Pearson's chi-square tests comparing the 2010 and 2022 surveys).

Age (years)	2010	2012	2014	2016	2018	2020	2022	χ^2^	*P*-value
7	75 (31.0)	95 (37.7)	80 (29.5)	93 (29.0)	61 (21.7)	58 (28.4)	39 (23.2)	10.13	0.002**
8	79 (29.2)	68 (26.2)	74 (30.3)	85 (29.4)	53 (23.7)	62 (26.7)	49 (24.0)	1.69	0.193
9	66 (26.3)	67 (29.9)	67 (24.8)	52 (20.2)	50 (20.1)	69 (31.9)	45 (23.9)	0.32	0.574
10	36 (14.1)	45 (18.8)	47 (16.7)	50 (22.6)	28 (11.9)	33 (16.0)	31 (14.8)	0.30	0.582
11	33 (11.6)	24 (11.5)	22 (9.6)	30 (11.8)	13 (6.1)	44 (19.9)	24 (12.4)	1.84	0.174
12	20 (7.3)	20 (9.9)	22 (8.6)	18 (7.1)	12 (5.6)	25 (14.0)	17 (9.2)	0.99	0.319
13	11 (4.3)	9 (3.3)	13 (5.9)	11 (5.8)	9 (4.5)	14 (7.4)	29 (16.1)	19.99	<0.001***
14	9 (3.6)	8 (3.6)	18 (7.6)	14 (5.7)	7 (3.3)	17 (10.1)	11 (7.3)	4.65	0.031*
15	5 (1.6)	5(2.4)	9(3.5)	5(2.8)	8(4.3)	7(4.1)	9(5.4)	5.72	0.017*

## Results

### Trends in the body mass index of children and adolescents, 2010–2022

The results on the trends in body mass index (BMI) among children and adolescents aged 7–15 from 2010 to 2022 (see Tables 8, 9) show significant differences in changes across gender and age groups. Among male children and adolescents, BMI did not exhibit a clear linear trend in the younger age group (7–9 years) (*P* > 0.05), however, starting at age 10, BMI showed a significant upward trend over the years (r_s = 0.082–0.189, *P* < 0.01), with the upward trend being particularly pronounced in the 12–15 age group (r_s = 0.156–0.189, *P* < 0.001). Changes in BMI among female children and adolescents exhibited divergent patterns: in the 7–10-year-old age group, there were no significant changes or a slight decline (e.g., r_s = −0.067, *P* = 0.005 for the 7-year-old group), whereas the 13–15-year-old age group showed a substantial increase (r_s = 0.156–0.197, *P* < 0.001). Compared to males, who showed a significant upward trend at ages 10–12, the onset of this significant increase in females occurred relatively later (ages 11–12), indicating that gender differences in the timing of puberty onset influence the trajectory of BMI changes. In addition, trends were not statistically significant in certain age groups, such as boys aged 7–9 and girls aged 8–10, which may be related to fluctuations in sample size or relatively stable growth and development during these age periods. Overall, between 2010 and 2022, the BMI of Chinese children and adolescents aged 7–15 showed a continuous upward trend after entering puberty. In particular, the risk of overweight and obesity significantly increased among older boys and girls, a dynamic trend that warrants close attention in public health policy.

**Table 8:** Trends in body mass index (BMI) among male children and adolescents by age group, 2010–2022 (*N* = 13,346; mean ± SD, kg/m^2^).

Age (years)	2010	2012	2014	2016	2018	2020	2022	r_s	*P* for trend
7	16.97 ± 4.93	16.93 ± 4.87	16.50 ± 4.20	17.08 ± 4.83	17.38 ± 4.62	16.58 ± 4.77	15.56 ± 2.93	−0.030	0.236
8	16.83 ± 4.82	17.31 ± 4.73	17.45 ± 5.08	17.26 ± 4.68	17.02 ± 4.07	16.73 ± 3.99	16.26 ± 3.72	−0.026	0.292
9	16.49 ± 3.88	17.05 ± 4.46	17.02 ± 4.49	17.15 ± 4.22	18.35 ± 4.96	16.79 ± 3.79	16.30 ± 2.91	0.042	0.106
10	16.38 ± 3.89	17.38 ± 4.41	17.50 ± 4.43	16.80 ± 4.06	18.84 ± 4.93	17.46 ± 4.28	16.92 ± 3.51	0.082	0.001**
11	17.08 ± 3.55	16.92 ± 3.52	17.11 ± 3.96	17.24 ± 4.05	18.70 ± 4.34	17.77 ± 3.72	17.38 ± 3.41	0.095	<0.001***
12	17.22 ± 3.80	17.33 ± 3.77	17.57 ± 3.47	17.50 ± 3.67	18.92 ± 4.20	18.49 ± 3.46	18.67 ± 4.08	0.156	<0.001***
13	17.25 ± 2.82	17.98 ± 3.90	17.81 ± 3.50	18.19 ± 3.30	18.97 ± 3.77	18.90 ± 3.37	18.75 ± 3.24	0.182	<0.001***
14	17.92 ± 2.93	18.68 ± 3.42	18.25 ± 2.62	18.62 ± 2.98	19.20 ± 3.37	19.13 ± 2.81	19.61 ± 3.03	0.189	<0.001***
15	18.45 ± 2.78	18.49 ± 2.60	19.03 ± 2.97	18.88 ± 2.85	19.19 ± 3.03	19.25 ± 2.82	19.47 ± 2.63	0.113	<0.001***

**Table 9:** Trends in body mass index (BMI) among female children and adolescents by age group, 2010–2022 (*N* = 14,409; mean ± SD, kg/m^2^).

Age (years)	2010	2012	2014	2016	2018	2020	2022	r_s	*P* for trend
7	17.33 ± 4.57	17.98 ± 4.92	17.22 ± 4.68	17.36 ± 5.02	16.59 ± 4.32	17.14 ± 4.45	16.76 ± 4.22	−0.067	0.005**
8	17.64 ± 4.66	17.42 ± 4.70	18.08 ± 5.37	17.51 ± 4.65	17.17 ± 4.28	17.22 ± 4.48	17.12 ± 4.14	−0.039	0.102
9	17.89 ± 5.18	18.43 ± 4.96	17.91 ± 4.76	17.48 ± 4.50	16.91 ± 4.20	18.43 ± 4.31	17.99 ± 4.42	−0.003	0.916
10	17.41 ± 4.39	18.00 ± 4.46	17.86 ± 4.27	18.12 ± 4.35	17.43 ± 3.88	18.10 ± 4.13	17.81 ± 4.42	0.016	0.516
11	17.74 ± 4.08	17.90 ± 4.55	17.79 ± 3.82	17.90 ± 3.70	17.47 ± 3.65	19.06 ± 4.53	18.53 ± 3.93	0.089	<0.001***
12	17.78 ± 4.08	18.33 ± 4.52	18.40 ± 3.90	17.92 ± 3.54	18.08 ± 3.28	19.20 ± 4.58	18.47 ± 3.64	0.079	0.002**
13	17.63 ± 3.63	18.19 ± 3.53	18.25 ± 3.79	18.21 ± 3.95	18.68 ± 3.27	19.01 ± 3.66	19.72 ± 4.30	0.156	<0.001***
14	18.30 ± 3.35	18.36 ± 3.54	19.03 ± 4.47	18.92 ± 3.89	18.98 ± 3.26	19.99 ± 3.87	19.15 ± 3.36	0.113	<0.001***
15	18.24 ± 2.94	18.64 ± 3.17	18.90 ± 3.23	19.03 ± 3.34	19.85 ± 3.07	19.82 ± 3.43	20.28 ± 3.44	0.197	<0.001***

### Prevalence of overweight among children and adolescents, 2010–2022

The results of the analysis of overweight prevalence among children and adolescents aged 7–15 from 2010 to 2022 (Tables 4, 5, and Figure 1) show significant differences in overweight rates across gender, age groups, and years. Overall, the overweight rate among male children and adolescents showed an upward trend in most age groups. Specifically, the overweight rates among male children and adolescents in the 8-, 13-, 14-, and 15-year-old age groups increased significantly over time (*P* < 0.05). Among them, the overweight rate in the 13-year-old group rose from 5.6% in 2010 to 14.2% in 2022 (*χ*^2^ = 16.73, *P* < 0.001), the 14-year-old group rose from 6.6% to 13.1% (*χ*^2^ = 10.86, *P* = 0.001), and the 15-year-old group rose from 5.4% to 6.7% (*χ*^2^ = 5.05, *P* = 0.025). Although the overweight rates in the 7-year-old and 9–12-year-old age groups fluctuated, these changes were not statistically significant (*P* > 0.05). Among female children and adolescents, the prevalence of overweight showed a significant upward trend only in the 15-year-old age group, rising from 4.2% in 2010 to 12.0% in 2022 (*χ*^2^ = 15.77, *P* < 0.001). Among male children and adolescents, the prevalence of overweight increased more markedly in the 8-year-old and 13–15-year-old age groups compared to females, indicating that the issue of overweight is becoming increasingly prominent among male children and adolescents. Furthermore, the prevalence of overweight reached a peak around 2018 across all age groups, a trend linked to socioeconomic developments and lifestyle changes at that time. Overall, between 2010 and 2022, the prevalence of overweight among children and adolescents aged 7–15 in China showed a significant upward trend in certain age groups—particularly among older male adolescents—suggesting that early screening and intervention for overweight remain essential.

**Figure 1:**
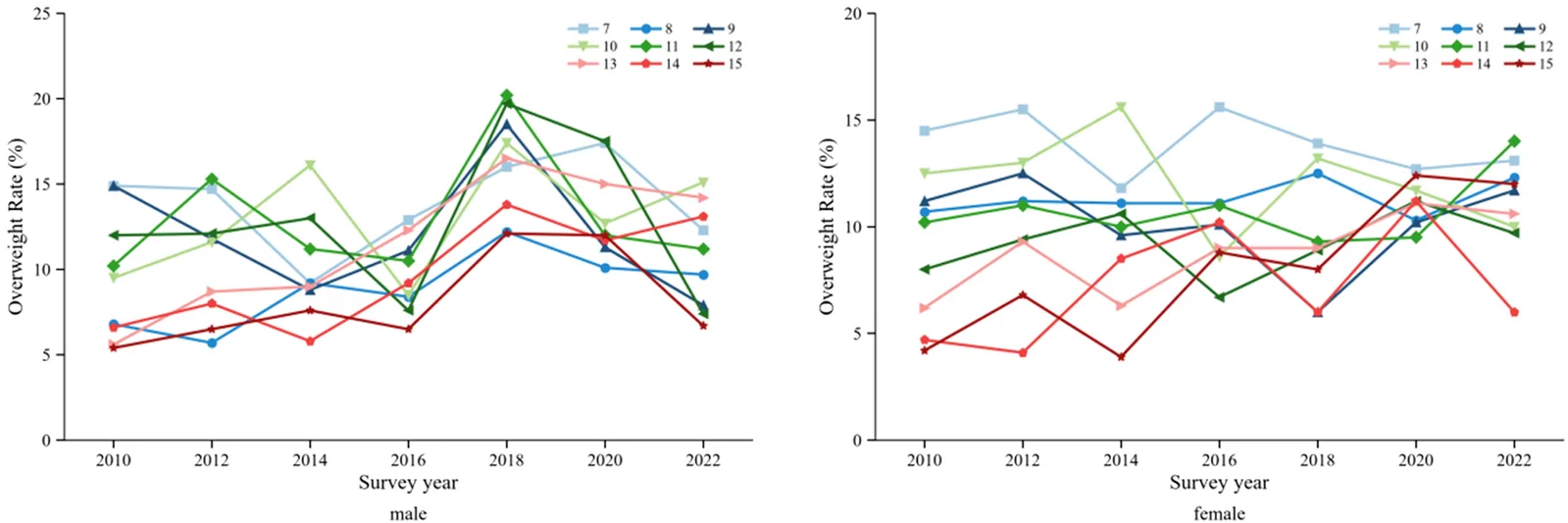
Prevalence of overweight among male and female children and adolescents, 2010–2022 (each line denotes an age group; the x-axis shows the survey year, and the figure directly shows the year-to-year trend in prevalence for each age group).

### Prevalence of obesity among children and adolescents, 2010–2022

Analysis of obesity prevalence among children and adolescents aged 7–15 from 2010 to 2022 (see Tables 6, 7 and Figure 2) reveals significant differences in obesity rates across gender, age groups, and years, with trends that are more complex than those for overweight. Among males, the obesity rate in the younger age group (7–8 years) showed a significant downward trend: the obesity rate in the 7-year-old group decreased from 23.8% in 2010 to 13.6% in 2022 (*χ*^2^ = 5.06, *P* = 0.025), and in the 8-year-old group, it fell from 20.0% to 12.6% (*χ*^2^ = 7.64, *P* = 0.006). In contrast, obesity rates in the 12- and 13-year-old groups showed a significant increase: the rate in the 12-year-old group rose from 4.7% in 2010 to 12.6% in 2022 (*χ*^2^ = 12.89, *P* < 0.001), and the rate in the 13-year-old group rose from 0.8% to 4.7% (*χ*^2^ = 7.85, *P* = 0.005). Although obesity rates in other age groups for males (9–11 years and 14–15 years) fluctuated, these changes were not statistically significant (*P* > 0.05). Among females, the obesity rate in the 7-year-old group showed a significant downward trend, decreasing from 31.0% in 2010 to 23.2% in 2022 (*χ*^2^ = 10.13, *P* = 0.002); whereas obesity rates in the 13-, 14-, and 15-year-old groups showed a significant increase: the 13-year-old group rose from 4.3% in 2010 to 16.1% in 2022 (*χ*^2^ = 19.99, *P* < 0.001), the 14-year-old group rose from 3.6% to 7.3% (*χ*^2^ = 4.65, *P* = 0.031), and the 15-year-old group rose from 1.6% to 5.4% (*χ*^2^ = 5.72, *P* = 0.017). Changes in other age groups among females were not statistically significant. Notably, obesity rates showed a marked downward trend among younger boys (ages 7–8) and 7-year-old girls, which may be related to early intervention and health education efforts targeting childhood obesity in recent years; conversely, obesity rates among both boys and girls in the middle-to-older age groups (ages 12–15) rose significantly, suggesting an increased risk of obesity during adolescence. Overall, between 2010 and 2022, the prevalence of obesity among children and adolescents aged 7–15 in China showed a downward trend in the younger age groups but rose significantly during adolescence, demonstrating distinct age-specific patterns. This necessitates the development of tailored intervention strategies for different age groups.

**Figure 2:**
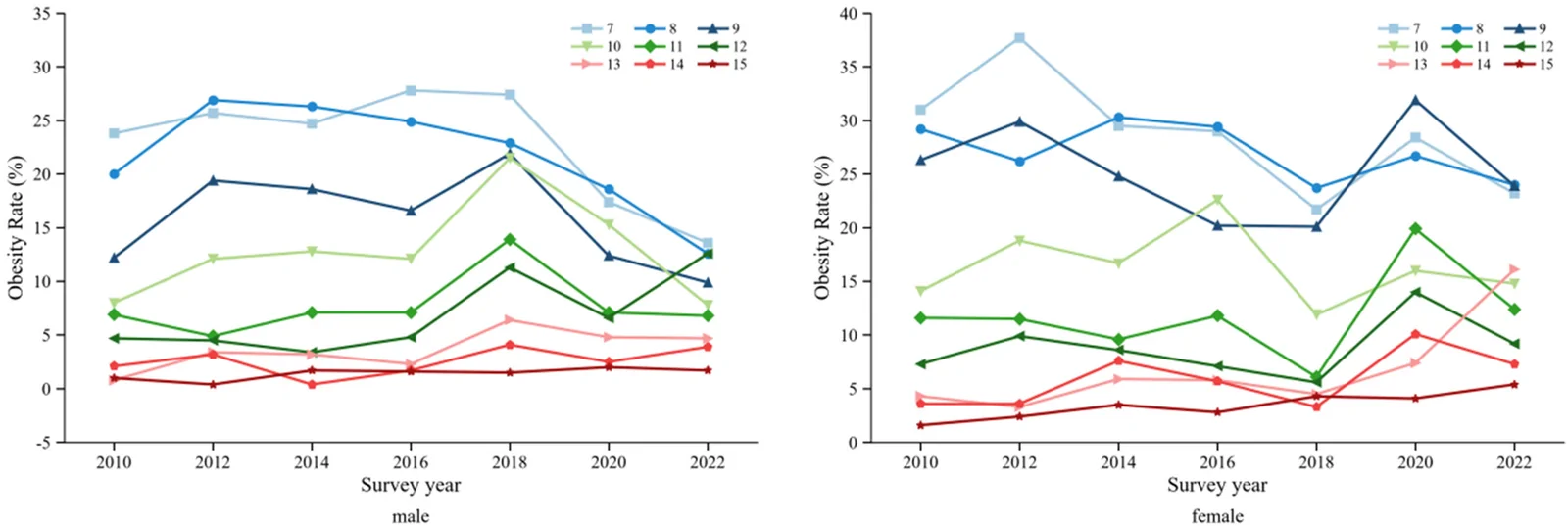
Prevalence of obesity among male and female children and adolescents, 2010–2022 (each line denotes an age group; the x-axis shows the survey year, and the figure directly shows the year-to-year trend in prevalence for each age group).

### Average annual percentage-point change in overweight and obesity prevalence among children and adolescents, 2010–2022

Based on the prevalence rates of overweight and obesity at each survey time point from 2010 to 2022, the average annual percentage-point change between adjacent time periods was calculated as follows: average annual percentage-point change = (prevalence rate in the later survey year—prevalence rate in the previous survey year)/number of years between surveys. Because prevalence was already expressed as a percentage, the result is reported in percentage points per year; the  × 100% term has been omitted to avoid double scaling (see Figure 3 and Tables 10, 11). In terms of the average annual percentage-point change in overweight prevalence, the 7- to 9-year-old male group showed relatively small fluctuations across all periods, with positive growth of 3.05 percentage points per year only between 2016 and 2018; the remaining periods mostly showed negative growth or minor fluctuations. The 10–12 age group showed the most significant increase between 2016 and 2018, with an annual growth of 5.10 percentage points, while the rates declined by 2.25 and 2.50 percentage points per year during the periods from 2014 to 2016 and from 2018 to 2020, respectively. Changes in the 13–15 age group were relatively stable, with an annual increase of 2.40 percentage points from 2016 to 2018, while fluctuations in other periods remained within ±1.0 percentage points. The annual variation in the overweight rate among females was generally smaller than that among males; for the 7–9 age group, changes in all periods mostly fell within ±1.0 percentage points. The 10–12 age group primarily experienced negative growth, with an annual increase of 0.85 percentage points observed only from 2016 to 2018. The 13–15 age group saw annual increases of 1.55 and 1.95 percentage points from 2014 to 2016 and from 2018 to 2020, respectively, while during other periods, the rate declined or fluctuated slightly. Looking at the annual changes in obesity rates, the rate among boys aged 7 to 9 increased by 2.65 percentage points each year from 2010 to 2012, after which it mostly declined, falling by 4.00 percentage points annually from 2018 to 2020. For boys aged 10 to 12, the obesity rate increased by 3.80 percentage points annually from 2016 to 2018, while the rate for those aged 13 to 15 showed little change. For girls aged 7 to 9, the obesity rate increased by 3.60 percentage points annually from 2018 to 2020 but decreased by 2.65 percentage points annually from 2020 to 2022. While the 10–12 age group saw an annual increase of 4.35 percentage points from 2018 to 2020, and the 13–15 age group saw annual increases of 0.65 and 1.60 percentage points from 2012 to 2014 and from 2018 to 2020, respectively. Overall, the annual changes in overweight and obesity rates among children and adolescents aged 7–15 exhibited distinct phased characteristics. Most age groups experienced a peak in positive growth between 2016 and 2018, while obesity rates among younger age groups of both males and females declined significantly between 2018 and 2020. During this period, the government vigorously implemented health intervention policies and achieved preliminary results. Among adolescents, particularly those aged 10 to 12, obesity rates rose rapidly during certain periods, indicating that this stage is a critical period of rapid weight gain. It is therefore necessary to continuously strengthen monitoring and interventions to curb the average annual percentage-point changes of overweight and obesity during this phase.

**Figure 3:**
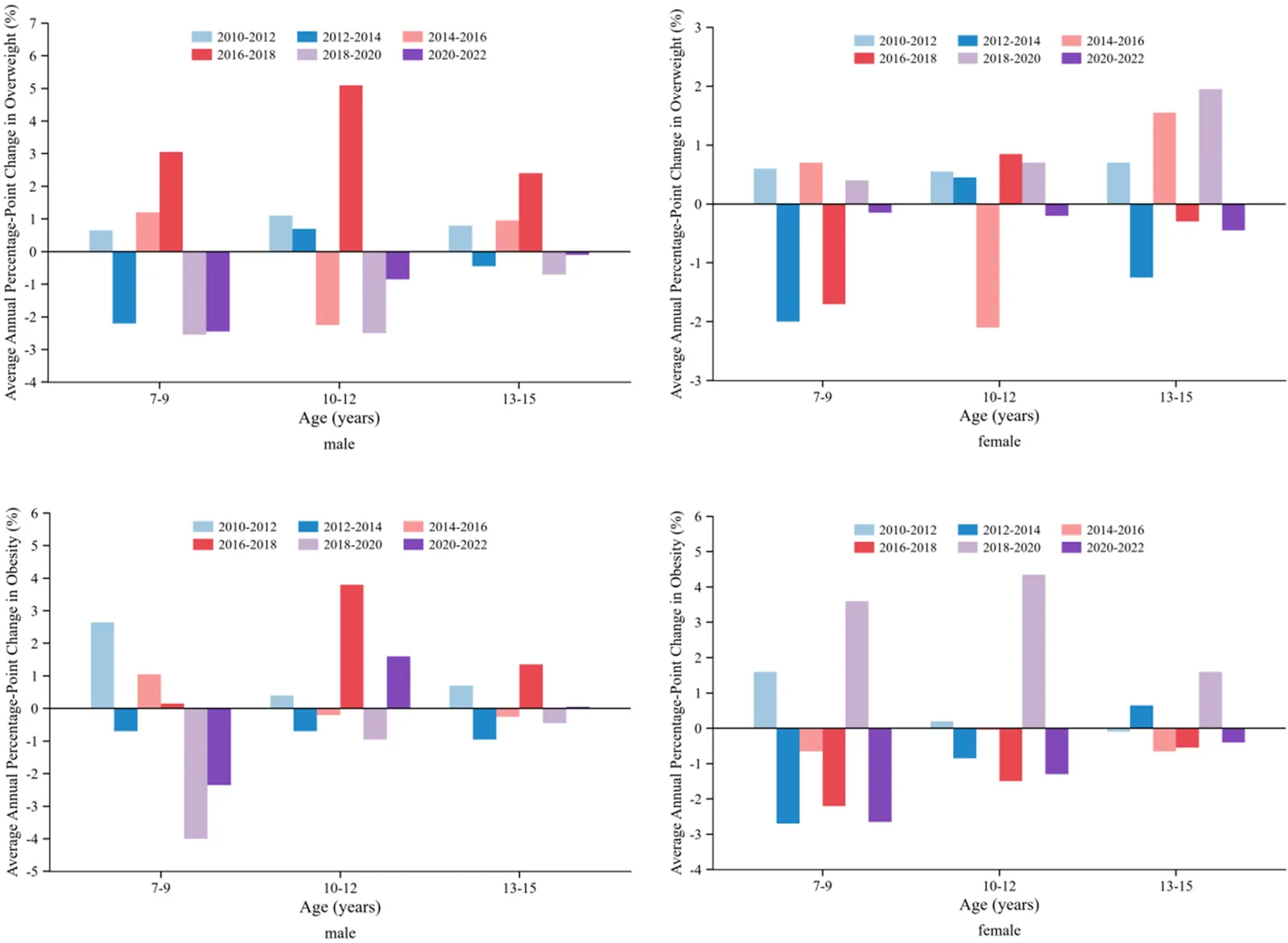
Average annual percentage-point changes in overweight and obesity prevalence among male and female children and adolescents by age group, 2010–2022.

**Table 10:** Average annual percentage-point changes in overweight and obesity among male children and adolescents by age group, 2010–2022 (percentage points per year; values are overweight/obesity).

Age (years)	2010–2012	2012–2014	2014–2016	2016–2018	2018–2020	2020–2022
7–9	0.65/2.65	−2.20/−0.70	1.20/1.05	3.05/0.15	−2.55/−4.00	−2.45/−2.35
10–12	1.10/0.40	0.70/−0.70	−2.25/−0.20	5.10/3.80	−2.50/−0.95	−0.85/1.60
13–15	0.80/0.70	−0.45/−0.95	0.95/−0.25	2.40/1.35	−0.70/−0.45	−0.10/0.05

**Table 11:** Average annual percentage-point changes in overweight and obesity among female children and adolescents by age group, 2010–2022 (percentage points per year; values are overweight/obesity).

Age (years)	2010–2012	2012–2014	2014–2016	2016–2018	2018–2020	2020–2022
7–9	0.60/1.60	−2.00/−2.70	0.70/−0.65	−1.70/−2.20	0.40/3.60	−0.15/−2.65
10–12	0.55/0.20	0.45/−0.85	−2.10/−0.05	0.85/−1.50	0.70/4.35	−0.20/−1.30
13–15	0.70/−0.10	−1.25/0.65	1.55/−0.65	−0.30/−0.55	1.95/1.60	−0.45/−0.40

## Discussion

### Overall trends in BMI: A slowing trend in younger age groups and an acceleration during adolescence

Based on longitudinal data from the China Family Panel Study covering 27,755 person-observations of children and adolescents aged 7–15 from 2010 to 2022, this study analyzes the dynamic trends in body mass index (BMI) for this population. The overall trend shows that BMI changes among children and adolescents of different genders and age groups are not synchronized. In the younger age group (7–9 years), neither boys nor girls exhibited a significant upward trend in BMI (*P* > 0.05). However, starting at age 10 for boys and age 11 for girls, BMI showed a significant upward trend over time (*P* < 0.01), which was particularly pronounced in the 12–15 age group. This is consistent with findings in the academic literature. Wang Yang and colleagues, through an analysis of data from the National Student Physical Fitness and Health Survey (2000–2019), found that the BMI of children and adolescents aged 7–18 in China has continued to rise; however, the rate of increase has slowed in recent years among younger age groups, while it has accelerated among older age groups (2), further indicating that age has a certain influence on BMI.A comparison with similar studies in the academic literature reveals that the findings of this study—which indicate an accelerating trend in BMI during adolescence—are consistent with the situation in most developed and middle-income countries worldwide. For example, the *World Obesity Report 2026* published by the World Obesity Federation shows that the obesity rate among school-aged children has risen from 4% in 1975 to nearly 20% in 2022, with particularly rapid growth in middle-income countries (3). Furthermore, a Swedish study indicates that the long-term trend of BMI increase during adolescence is more strongly associated with the risk of cardiovascular disease in adulthood than is the case during childhood (4). An analysis of the reasons behind the trend of accelerated BMI increase during adolescence and a slowing trend in younger age groups among children and adolescents aged 7–15 reveals that, on the one hand, policy interventions at the government level have influenced the overall trend in BMI among this population. For example, China's Nutrition Improvement Program for Students in Rural Compulsory Education, launched in 2011, along with a series of subsequent health-related policies, has not only improved students' nutritional status but also raised public awareness of the prevention and control of overweight and obesity among students (5); furthermore, the state has successively introduced a series of policies, such as the ‘Healthy China 2030’ Outline and the Implementation Plan for the Prevention and Control of Obesity in Children and Adolescents, which not only guide and promote the dissemination of health knowledge at the macro level but have also significantly increased parents' attention to weight management for young children (6). On the other hand, the accelerated rise in BMI among adolescents can be attributed to increased appetite induced by hormonal changes, reduced physical activity resulting from increased academic pressure, and a rise in the prevalence of unhealthy dietary behaviors (7). Adolescent sport participation attitudes are closely associated with physical activity involvement and physical health and are shaped by health communication through sports social media, self-efficacy and social support (8). Existing research indicates that boys enter the phase of significant BMI increase at an earlier age than girls, a pattern consistent with the timing of puberty onset. A study by Zhao Ruilan et al. on school-aged children in Beijing showed that the age at which boys' height growth rate peaks is 12 years, while for girls it is 10 years; boys reach their peak height growth rate two years later than girls, but their peak growth rate is higher than that of girls (9), resulting in a longer window of rapid BMI increase for boys during the middle and late stages of puberty.

### Gender differences in overweight prevalence: A pattern of early onset in males and late onset in females

Compared to the overall trends in body mass index (BMI), this study found that the trends in overweight prevalence across different genders and age groups were more complex, and the differences between groups were statistically significant. Specifically, among male children and adolescents, the prevalence of overweight in the 8-, 13-, 14-, and 15-year-old age groups showed a significant upward trend over the survey years (*P* < 0.05). Among them, the prevalence of overweight in the 13-year-old age group rose from 5.6% in 2010 to 14.2% in 2022, an increase of more than 150%;Among females, only the 15-year-old age group showed a significant upward trend in the overweight rate, rising from 4.2% in 2010 to 12.0% in 2022. These results indicate that the problem of overweight is more pronounced among male adolescents than among their female peers of the same age, and that it is spreading from younger to older age groups. This age group is at a critical stage of transition from childhood to preadolescence; overweight conditions established during this period typically persist into adolescence and even adulthood. This conclusion is consistent with the findings of numerous existing academic studies. For example, a survey of Vietnamese elementary school students conducted by Le Giang Ba and colleagues found that the obesity rate among boys was approximately twice that of girls (10). A study by Rodriguez Alina et al., based on a sample of 19,634 early adolescents, further confirmed that male adolescents have a significantly higher risk of becoming overweight or obese in early puberty than their female peers (11). However, due to factors such as heterogeneity in weight classification criteria, differences in socioeconomic environments, and shifts in the age distribution of the population, the prevalence of overweight across different age groups does not exhibit a monotonic increase but rather a fluctuating upward trend. The reasons for this are twofold: on the one hand, there are significant differences in how boys and girls perceive their body shape during adolescence. Females pay significantly more attention to their body shape and generally tend to maintain a slim figure through dieting; they even perceive a need to lose weight even when their weight is within the normal range. In contrast, males are less sensitive to perceptions of being overweight and prefer to consume high-calorie snacks and sugary beverages, resulting in a more pronounced imbalance between total energy intake and expenditure compared to females (12). On the other hand, changes in the socioeconomic environment have influenced weight trends among children and adolescents. Since 2018, the number of mobile internet users in China has exceeded 1 billion, and online food delivery services have rapidly become widespread. While this has reduced residents' physical activity levels, it has also driven a continuous rise in energy intake. Furthermore, studies have shown that prolonged screen time significantly increases the risk of obesity among adolescents (13). These characteristics have also led to a relative peak in the prevalence of overweight among male adolescents in most age groups, indicating a need to place greater emphasis on interventions targeting overweight issues among male adolescents, strengthen health behavior education, and optimize the family dietary environment.

### Age-specific trends in obesity prevalence: A cross-point where rates improve among younger age groups and worsen among older ones

The trends in obesity prevalence are more complex than those for overweight and exhibit distinct age-specific patterns. This is primarily manifested as follows: obesity rates among younger males and females show a significant downward trend; among 7-year-old boys, the obesity rate fell from 23.8% in 2010 to 13.6% in 2022, and among 8-year-old boys, it dropped from 20.0% to 12.6%; among 7-year-old girls, the rate decreased from 31.0% to 23.2%.In contrast, obesity rates among adolescents in older age groups showed the opposite trend: for boys, the rate in the 12-year-old group rose from 4.7% to 12.6%, and in the 13-year-old group from 0.8% to 4.7%; for girls, the rate in the 13-year-old group jumped from 4.3% to 16.1%, and in the 14-year-old group from 3.6% to 7.3%.A study by Cheng Wenlin and colleagues involving 10,939 adolescents aged 10–15 from 2010 to 2016 found that the rate of change in overweight and obesity among boys was lower from 2012 to 2014 than from 2014 to 2016, with the growth rate slowing significantly in the latter period (14). At the international level, research by the NCD Risk Factors Collaboration indicates that global obesity rates among children and adolescents continued to rise after 2010, but there was significant heterogeneity in age distribution across different regions, with the trend toward obesity in older age groups particularly pronounced in high-income countries (15). On the one hand, the positive changes in obesity rates among younger children may be at least partly attributable to systematic policy interventions implemented at the national level since 2010, although this observational study cannot establish causation. The ‘Expert Consensus on Obesity Prevention and Control Among Chinese Residents’ clearly states that the Rural Compulsory Education Student Nutrition Improvement Program launched in 2011, the ‘National Student Physical Fitness Standards’ promulgated in 2014, and the ‘Childhood Obesity Prevention and Control Action’ jointly carried out by multiple departments in 2019—through comprehensive measures such as health education in kindergartens and early elementary grades, nutritionally balanced meals in school cafeterias,one hour of daily physical activity, may have contributed to curbing the rising trend in obesity rates among young children. An analysis by Du Wenwen and other scholars of BMI trends among Chinese children aged 6–17 from 1982 to 2016 also confirms that the rate of increase in children's BMI has slowed since 2012 (16). At the same time, widespread media coverage of the dangers of obesity and improvements in parents' health literacy have prompted families to pay greater attention to weight management for young children (17). On the other hand, while existing obesity prevention and control measures can have some effect on young children, they have not yet been able to effectively prevent childhood obesity from persisting into adolescence. External controls through families and schools during early childhood can temporarily curb excessive weight gain. However, once children and adolescents enter puberty, the combined effects of snacking outside of school, consumption of sugar-sweetened beverages, insufficient sleep, increased academic pressure, and reduced physical activity lead to a concentrated release of previously accumulated obesity risks during adolescence (18). The results of this study also show that the obesity rate among 13-year-old girls reached 16.1% in 2022, far exceeding the 4.7% rate among boys of the same age. This change is closely linked to hormonal fluctuations during adolescence; an increase in body fat percentage among females during this stage is a natural physiological trend, which also corroborates the reality that female adolescents' participation rates in physical activity are significantly lower than those of their male peers. In response, greater attention must be paid to fostering healthy exercise habits among children and adolescents entering puberty, providing support for their mental health, and preventing body image anxiety from leading to unhealthy weight-loss behaviors among female children and adolescents. Childhood obesity is frequently accompanied by metabolic comorbidities; a recent meta-analysis reported a high prevalence of dyslipidemia among Chinese children and adolescents (19).

### Phased characteristics of average annual percentage-point changes: alternation between policy-sensitive and risk-accumulation periods

This study analyzed the average annual percentage-point changes of overweight and obesity rates across adjacent survey periods and found that the annual changes in these rates exhibit distinct phased characteristics. This is primarily reflected in the fact that from 2016 to 2018, the average annual percentage-point changes of overweight and obesity reached phased peaks in most age groups. Specifically, the annual increase in overweight rates among 10- to 12-year-old boys reached 5.10 percentage points, while the annual increase in obesity rates for the same age group was 3.80 percentage points; the annual increase in obesity rates among 10- to 12-year-old girls also reached as high as 4.35 percentage points. These divergent patterns during this phase are consistent with the conclusions drawn from relevant domestic and international studies. In an analysis of trends in overweight and obesity among Chinese children and adolescents from 2000 to 2019, some scholars pointed out that from 2014 to 2019, the growth rate of overweight prevalence in younger age groups declined significantly, while the growth rate of obesity prevalence in older age groups actually accelerated, reflecting a pattern of 'slowing among younger age groups and accelerating among older age groups' (2). Other scholars, based on data from the China Health and Nutrition Survey, noted that between 2000 and 2018, the dietary patterns of children and adolescents underwent significant changes: the proportion of energy derived from fat continued to rise, while that from carbohydrates declined; meanwhile, the increased frequency of takeout consumption may have (20) may have contributed to the spread of fast-food-style eating habits—characterized by high sugar, fat, and salt content—among adolescents. From 2015 to 2018, the number of takeout users in China surged from less than 100 million to 400 million. Moreover, takeout consumption is often accompanied by increased screen time, which further diminishes the perception of satiety and the ability to self-regulate energy intake. On the other hand, the deep integration of digital entertainment may have reduced (21)average annual percentage-point change of obesity among 7- to 9-year-old boys decreased by 4.00 percentage points, while that among 7- to 9-year-old girls decreased by 2.65 percentage points, which may reflect, at least in part, the synergistic effects of public health policy interventions and a societal consensus on health. In 2018, the Ministry of Education and seven other departments jointly issued the ‘Implementation Plan for Comprehensive Prevention and Control of Myopia Among Children and Adolescents,’ which, for the first time, incorporated measures such as increasing the duration of outdoor activities and regulating the use of electronic devices into mandatory assessment indicators. This made the early elementary school years the primary focus of policy implementation, thereby contributing to the downward trend in the annual obesity growth rate. It is evident that weight management strategies should not be limited to correcting individual behavior but must also address the ‘obesogenic environment.’ Multidimensional, systematic interventions should be implemented through measures such as regulating food promotion practices on food delivery platforms targeting adolescents, optimizing the supply of healthy foods near schools, and establishing guidelines for the average daily usage time of digital devices among children.

### Implications of the research findings for public health policy

The trends revealed in this study—namely, a narrowing of BMI variation among younger children and adolescents and a widening of variation among older children and adolescents—can provide clear practical guidance for the targeted formulation of weight management policies for this population. First, the increase in BMI and the prevalence of overweight and obesity among children and adolescents is primarily concentrated during adolescence, with more significant increases observed in boys after age 10 and in girls after age 12. Consequently, the intervention window for obesity prevention and control can be shifted from the traditional focus on young children to the upper elementary grades before adolescence, and extended through the junior high school years. Currently, most health promotion programs target lower elementary grades as their primary audience, with significant gaps in coverage for junior high school students—despite adolescence being a critical period marked by rapid weight gain and the solidification of unhealthy lifestyle habits. International comparative experiences suggest that embedding health-literacy components within school curricula may help reduce the cumulative risk of overweight and obesity among adolescents; several countries have integrated such modules into school-based programmes (22). In response, China's education and health authorities could consider introducing specialized weight management courses during junior high school, organically integrating nutrition education, physical activity, and mental health content, and establish a three-tiered collaborative mechanism involving ‘vice principals for health’ or 'school nurses–homeroom teachers–parents.' Second, the rise in overweight rates among male children and adolescents in certain older age groups is more pronounced, occurring at an earlier age and affecting a broader range of areas, making it urgent to design gender-sensitive intervention strategies. Research shows that overweight and obese children exhibit functional abnormalities in brain regions involved in reward processing and inhibitory control, and that males exhibit stronger responses to visual cues associated with high-calorie foods (23)^.^ In response, for this group of male children and adolescents, group intervention programs integrating physical activity and gamification could be developed by leveraging their preferences for sports events and video games. Third, the positive trend in obesity rates among younger children may be attributable to recent comprehensive measures, including collaboration between schools and families, improvements to school cafeterias, and restrictions on high-sugar snacks on school campuses. However, it remains to be seen whether this improvement will be sustained; it is suggested that successful approaches for younger children—such as a unified school food distribution system (5) and systematically summarized best practices—be extended to older age groups of children and adolescents. Furthermore, this study shows that obesity rates among certain age groups during adolescence continued to rise between 2018 and 2020, indicating that current policies may be insufficient to effectively prevent weight issues from persisting into adolescence. In response, future policy-making should shift from fragmented interventions to a collaborative health governance model spanning the entire life cycle and involving multiple sectors. It should adhere to the concept of ‘multidisciplinary collaborative diagnosis and treatment’ proposed in the Chinese Guidelines for the Diagnosis and Treatment of Obesity (2024 Edition) (24)^,^ to help departments such as education, sports, health, and market regulation establish joint working groups. Examples include: sports departments opening free sports facilities on weekends; communities establishing youth sports clubs; the media reducing targeted advertising of high-calorie foods to adolescents; and the healthcare system incorporating BMI screening into annual physical examinations for primary and secondary school students, while implementing tiered management and personalized prescription-based interventions for overweight and obese students.

## Limitations

This study has several limitations that should be acknowledged. First, height and weight in CFPS may have been self- or proxy-reported rather than objectively measured in some waves, and the mode of measurement was not uniform across waves and could not be verified, potentially introducing measurement error. Second, because CFPS is a rotating panel with changing composition across waves, the analytical sample comprises repeated cross-sections rather than a fixed cohort; attrition and non-response across waves may therefore introduce selection bias into the trend estimates. Third, although CFPS provides information on urban/rural residence, region, socioeconomic status, diet, physical activity, and screen time, these potential determinants were not examined in the present analysis. Fourth, as an observational study, the reported trends reflect associations rather than causal effects of specific policies or behavioral changes. Fifth, because repeated observations from the same individuals are not independent and the analyses did not apply CFPS sampling weights, the prevalence estimates may not be strictly nationally representative. Sixth, the coronavirus disease 2019 (COVID-19) pandemic may have altered behaviors and thus affected the 2020 survey wave in ways not captured here. Finally, the height and weight cut-offs used to remove physiologically implausible values (0.8–2.2 m; 15–150 kg) were applied without an additional extreme-value algorithm.

In summary, this study reveals the dynamic evolution of BMI among Chinese children and adolescents aged 7–15 from 2010 to 2022, as well as trends in the prevalence of overweight and obesity. The results show that the prevalence of obesity among younger children has been declining, but the problems of overweight and obesity among adolescents continue to worsen, with significant gender and age-specific variations. The conclusions drawn from this study provide a scientific basis for formulating stage-specific and population-specific weight management strategies for children and adolescents. Future research and public health practices should further advance the development of a lifelong health monitoring system and the implementation of multidimensional, comprehensive interventions to effectively curb the rise in obesity among children and adolescents.

## Data Availability

Publicly available datasets were analyzed in this study. This data can be found here: http://www.isss.pku.edu.cn/cfps/ (China Family Panel Studies [CFPS]).
